# An engineered human conjunctival-like tissue to study ocular surface inflammatory diseases

**DOI:** 10.1371/journal.pone.0171099

**Published:** 2017-03-01

**Authors:** Laura García-Posadas, Laura Soriano-Romaní, Antonio López-García, Yolanda Diebold

**Affiliations:** 1 Ocular Surface Group, Institute for Applied Ophthalmobiology (IOBA), University of Valladolid, Valladolid, Spain; 2 CIBER-BBN (Biomedical Research Networking Center on Bioengineering, Biomaterials and Nanomedicine), Valladolid, Spain; Kyoto Daigaku, JAPAN

## Abstract

The aim of this study was to develop a three-dimensional model of the human conjunctiva that can be used to perform physiology and pathophysiology experiments. Fibrin-based matrices (derived from human plasma or plasma cryoprecipitate) were used as scaffolds, and primary cells were obtained from conjunctival tissue. Conjunctival constructs were analyzed by immunofluorescent staining and scanning electron microscopy and cell proliferation was measured with alamarBlue^®^ assay. After characterizing the constructs, four different experimental conditions were analyzed in cryoprecipitate matrices: controls, air-lifted cultures (to increase cell stratification), partially desiccated cultures (to mimic dry eye disease), and IL-13-treated cultures (to mimic allergy). Constructs were stained with hematoxylin/eosin to observe changes in morphology. High molecular weight glycoconjugates were identified by HPA staining. MUC5AC and IL-6 secretion was evaluated by ELISA. The fibrin-based matrices supported conjunctival cell growth. Epithelial cells grew on the surface of the scaffolds and underwent stratification that increased over time. These cells had microvilli, which suggests cell polarization and functionality. Fibroblasts were integrated in the scaffold and showed elongated shape. Compared to controls, air-lifted construct had increased epithelial stratification and upregulated MUC5AC secretion. Increased MUC5AC secretion also occurred in partially desiccated and IL-13-treated cultures. The inflammatory status of cells was evaluated by IL-6 levels which were increased in air-lifted and partially desiccated cultures, but not in IL-13-treated ones. In conclusion, we have developed a new three-dimensional model of human conjunctiva that can be used to study ocular surface inflammatory diseases.

## Introduction

Inflammatory ocular surface diseases are very prevalent among the global population. Patients demand more efficacious, new treatments for their diseases and, at the same time, governments and pharmaceutical companies are concerned about the cost of the research needed to develop new drugs [1]. The increasing use of three-dimensional models has shown their utility in decreasing research costs by providing more reliable results and reducing the use of animals in research [2].

The conjunctiva is the mucous membrane that covers the inner surfaces of the eyelids and extends to the cornea. It is involved in different ocular surface diseases, playing an active role in the pathophysiology of common conditions such as dry eye disease [3], Sjögren’s syndrome [4], and allergic conjunctivitis [5], among others. There are several factors associated with these diseases that induce inflammation in conjunctival tissue. For instance, in dry eye disease, desiccation and other stimuli can trigger the inflammatory response [6]. In allergies, the presence of large amounts of some Th2-type cytokines, for example interleukin 13 (IL-13), can cause inflammatory signs such as mucous hypersecretion [7].

To date, the majority of *in vitro* investigations concerning the conjunctival tissue have been carried out using monolayer culture techniques that do not recapitulate the complexity of the whole tissue. Interactions between different cells and between cells and the extracellular matrix cannot be studied in those types of *in vitro* models. Consequently, there is a large gap between our knowledge of cellular processes and our understanding of the biology at the tissue level [8].

The conjunctiva is comprised of an epithelium and the underlying stroma to which it is anchored. Human conjunctival epithelial cell layers varies from two to ten, depending on the anatomic area. Bulbar conjunctiva usually shows two to four cell layers [9,10]. The most abundant cells in the conjunctiva are the epithelial cells (including goblet cells, specialized in mucin secretion) and the fibroblasts, all of which are active participants in the inflammatory process [11–13]. Therefore, a good *in vitro* model to study conjunctival diseases should contain, at least, a three-dimensional scaffold representing the stroma with fibroblasts inside, and a stratified epithelium anchored to that scaffold. In addition, the epithelium should maintain the mucus-secretion capacity that it exhibits *in vivo*.

Tissue engineering has provided the necessary knowledge to combine biomaterials and different cell types to produce tissue equivalents that can be used for research or transplantation. In the last few years, some attempts to elaborate conjunctival equivalents have been reported, most of them focused on clinical use [14–19]. Therefore, their main goal was not to mimic the human conjunctiva in a way that is useful to study it *in vitro*, but to make it suitable for transplantation. There are a few three-dimensional models of the conjunctiva that can be used to perform *in vitro* experiments, although not all of them include stroma and fibroblasts [20]. In this study, we have developed a novel three-dimensional model of inflamed human conjunctiva and used partial desiccation and IL-13 treatment to mimic some of the features observed in conjunctival diseases and to test the validity of the model. A vast majority of *in vitro* research focused in the conjunctiva is performed in squamous stratified epithelial cell lines, or isolated primary goblet cells or fibroblasts. Those models lack the three-dimensional structure that allow cells to polarize, and the cell-cell interaction that helps cell differentiation. This is the first time that such a complex model of human conjunctiva has been developed and aimed to *in vitro* studies, and the cell functionality has been tested in inflammatory conditions relevant to prevalent eye diseases.

## Materials and methods

### Human conjunctival tissues

Human bulbar conjunctival tissues were obtained from the Barraquer Eye Bank of Barcelona (Spain). None of the transplant donors were from a vulnerable population and all donors or next of kin provided informed consent that was freely given. This study was in strict accordance with the tenets of the Declaration of Helsinki and Spanish Regulations concerning the use of human tissues for biomedical research, and had approval of the Institutional Review Board of the University of Valladolid. The conjunctivas were carefully isolated from corneoscleral buttons extirpated from cadaveric donors (n = 20).

### Isolation and culture of conjunctival cells

Conjunctival cells were cultured as previously described [21]. Briefly, bulbar conjunctival tissue was carefully cut in small pieces of approximately 1mm^2^, and placed in 12-well plates containing 350 μl of culture medium. Fibroblasts were cultured in supplemented Dulbecco’s Modified Eagle Medium/Nutrient Mixture F-12 (DMEM/F12, Invitrogen, Inchinnan, UK) culture medium supplemented with 2.5 μg/ml fungizone, 5 000 units/ml of penicillin/streptomycin, and 10% human serum. Epithelial cell culture medium was composed of DMEM/F12 supplemented with 2.5 μg/ml fungizone, 5 000 units/ml of penicillin/streptomycin, 1 μg/ml insulin, 0.5 μg/ml hydrocortisone (Sigma-Aldrich, St. Louis, MO, USA), 2 ng/ml epidermal growth factor, and 10% human serum (Lonza Group Ltd., Basel, Switzerland), all from Invitrogen unless otherwise indicated.

After 7–10 days in culture, cells were trypsinized and passaged. Fibroblasts and epithelial cells were isolated by differential trypsinization, as previously described [21]. Fibroblasts in passage 3 and epithelial cells in passage 1 were used for all the experiments.

Both fibroblasts and epithelial cells were maintained in standard conditions (humidified atmosphere of 5% CO_2_ at 37°C), and the medium was changed every other day.

### Fibrin scaffold preparation

Fibrin-based matrices were used as scaffolds to engineer the conjunctival-like tissue. They were prepared from human fresh frozen plasma or plasma cryoprecipitate, obtained from the Blood Tissue Bank *Centro de Hemoterapia y Hemodonación de Castilla y León* (CHEMCYL, Valladolid, Spain). Scaffolds were produced by mixing 400 μl/ml fresh frozen plasma or 333 μl/ml cryoprecipitate with 40 μl/ml tranexamic acid (Rottapharm, Valencia, Spain) and 40 μl/ml calcium chloride (Braun, Barcelona, Spain), all diluted in DMEM/F12 culture medium.

### Cell seeding of fibrin scaffolds

Fibroblasts were grown inside the fibrin-based scaffolds. The cells were incorporated into the scaffolds as they were prepared, adding 100 000 cells/ml to the plasma or cryoprecipitate matrix at the time of preparation. Epithelial cells were seeded onto the surface of fibrin scaffolds 24 h after fibrin polymerization, at a cell density of 100 000 cells/cm^2^.

### Cell proliferation assays

On days 3 and 7, we used the non-toxic fluorescence dye alamarBlue^®^ (AbD Serotec, Oxford, UK) to evaluate cell proliferation in the conjunctival constructs. Matrices were incubated in medium with 10% alamarBlue^®^ for 6 h. After incubation, that medium was recovered, and the fluorescence was measured at 560 nm excitation and 590 nm emission wavelengths, using the SpectraMax M5 fluorescence plate reader (Molecular Devices, Sunnyvale, CA, USA). Four independent experiments were performed.

### Scanning Electron Microscopy (SEM)

We evaluated fibrin-based conjunctival constructs by SEM. Matrices were cut and fixed with 2.5% glutaraldehyde (Sigma-Aldrich), dehydrated in a graded ethanol series, and dried in a critical point drier. Samples were then viewed in an ESEM QUANTA 200 FEG scanning electron microscope (FEI Company, Hillsboro, OR, USA).

### Experimental conditions

**Fig 1:**
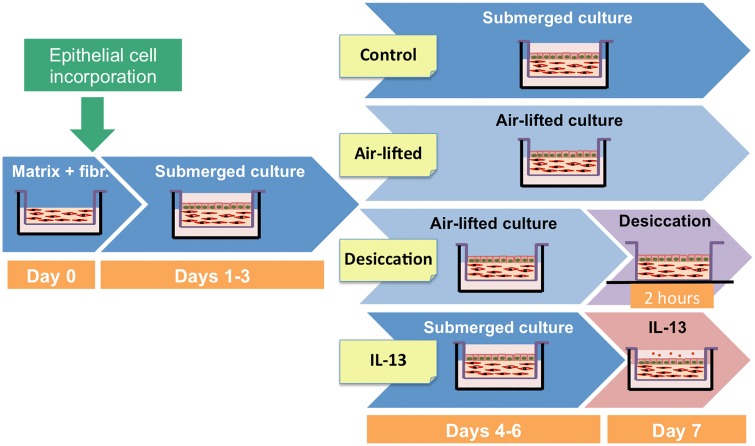
Experimental condition protocols. Fibr, fibroblasts.

We used four different experimental conditions in this study (Fig 1). Conjunctival constructs cultured for 7 days in standard submerged conditions served as controls. In addition, other constructs were cultured submerged for 3 days and then for 3 days in air-lifted conditions to stimulate cell stratification. To simulate some of the features found in dry eye patients, a partially desiccating condition was also used, placing the constructs without medium in the culture hood for 2 h, what cause partial drying. Finally, to simulate an allergic response, the cultures were stimulated with 20 ng/ml IL-13 (PeproTech, London, UK) for 24 h.

### Histological processing

At different time points, complete constructs of fibrin scaffolds with conjunctival cells were fixed with 4% formaldehyde after rinsing three times in phosphate buffered saline (PBS). Fixed samples were processed and embedded in paraffin. Five-μm sections were cut and collected on poly-L-lysine coated slides that were kept at -80°C until used. Sections were stained with hematoxylin and eosin (H/E) and viewed under an Eclipse TS100 (Nikon, Tokyo, Japan) light microscope.

Matrix thickness was measured in stained sections using the Eclipse TS100 microscope. Ten different measures (not including epithelium thickness) were taken in each experiment from five randomly taken pictures. Six different experiments were performed.

### Immunofluorescence and lectin-binding assays

Slides were deparaffinized in xylene and rehydrated in decreasing concentrations of ethanol. After antigen retrieval with 0.01% trypsin, sections were blocked in PBS with 4% donkey serum (Sigma-Aldrich) and 0.03% Triton X-100. Primary antibody against CK19 (Dako, Glostrup, Denmark; 1:50 dilution), a marker of conjunctival epithelial cells, was applied overnight at 4°C. Primary antibody against Ki67 (Abcam, Cambridge, UK; 1:50 dilution), a marker of cell proliferation, was applied for 1 h at 37°C. After washing 3 times with PBS, AlexaFluor^®^ 488 or AlexaFluor^®^ 647 secondary antibodies (Invitrogen) were applied for 1 h at room temperature (RT). Negative controls included the omission of primary antibodies and were included in each experiment. Specificity of antibodies had been previously tested in our laboratory. Nuclei were counterstained with Hoechst dye (Sigma-Aldrich) in all slides.

We identified glycoconjugates by lectin staining. The agglutinin obtained from *Helix pomatia* (HPA) was used at 1:500 dilution, for 30 min at RT. HPA binds to high molecular weight glycoconjugates secreted by goblet cells. Nuclei were counterstained with Hoechst dye. The preparations were viewed under a Leica DMI 6000B epifluorescence microscope (Leica Microsystems, Wetzlar, Germany).

### Secreted protein measurement by Enzyme-Linked Immunosorbent Assay (ELISA)

We measured the levels of secreted MUC5AC and IL-6 proteins by ELISA of culture supernatants recovered from the constructs exposed to the four experimental conditions. For these analyses, we used commercial ELISA kits (MUC5AC ELISA from Shanghai Yehua Biological Technology Co., Ltd., Shanghai, China; IL-6 ELISA from Diaclone, Besançon, France) and followed the manufacturer’s instructions.

### Statistical analysis

We used the Statistical Package for the Social Sciences software (SPSS 15.0, SPSS Inc., Chicago, IL, USA) for statistical analyses. Data were calculated as means ± standard error of the means. One-way analysis of variance (ANOVA) was used for comparison of mean values after assuring equality of variance (Levene’s test). After that, pairwise comparisons (Tukey test) were performed. Differences were considered to be significant when p ≤ 0.05.

## Results

### Fibrin-based matrices supported conjunctival cell growth

**Fig 2:**
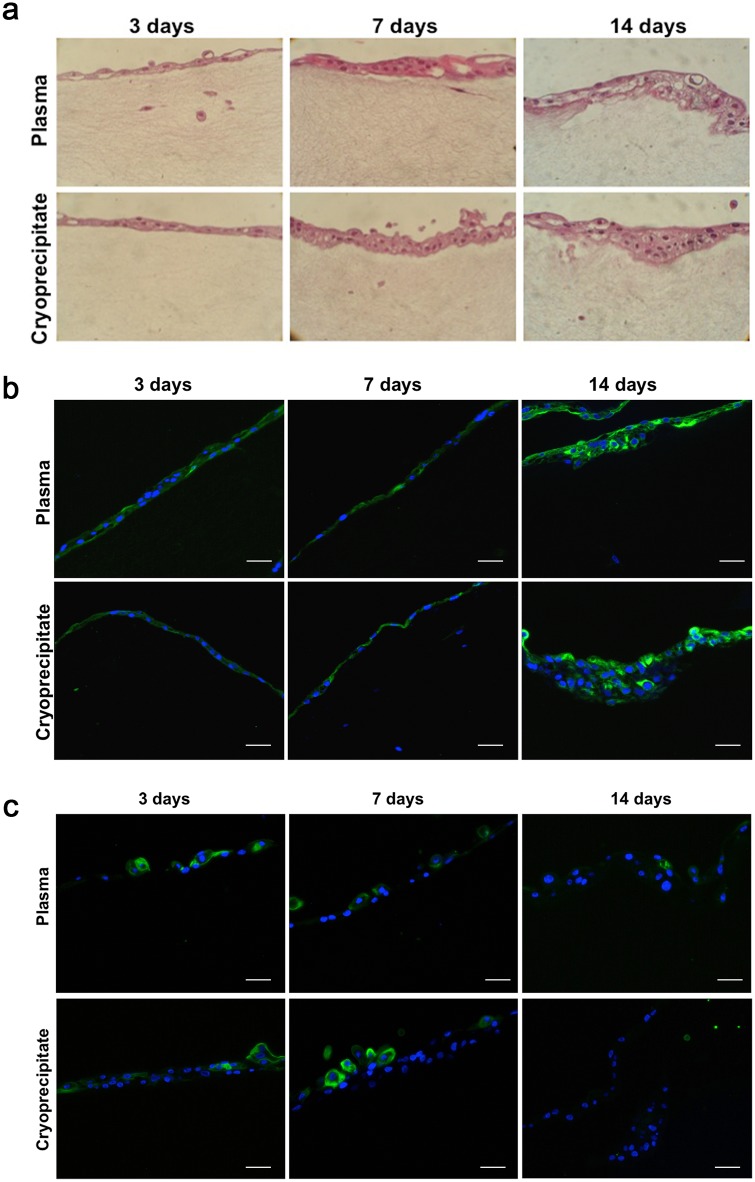
Conjunctival cells grown in and on fibrin scaffold constructs. **(a)** Cell seeded constructs derived from plasma or cryoprecipitate were stained with hematoxylin/eosin at days 3 (left), 7 (middle), and 14 (right). The number of epithelial cell layers increased over time. Magnification: X20. **(b)** Representative microphotographs of CK19 staining in the constructs. Epithelial cells maintained CK19 staining (green) up to day 14 in both plasma (top) or cryoprecipitate (bottom) scaffolds. Cell nuclei are stained with Hoechst dye (blue). Scale bar: 50 μm. **(c)** Representative microphotographs of HPA staining in the conjunctival constructs. Epithelial cells cultured on the surface of fibrin scaffolds produced mucins (green) at days 3 (left) and 7 (middle), but they did not produce them at day 14 (right). Cell nuclei are stained with Hoechst dye (blue). Scale bar: 50 μm.

Fibrin matrices supported human conjunctival cell growth. Both fibroblasts and epithelial cells were maintained in the scaffolds for at least 21 days as observed in H/E stained sections (Fig 2a). Conjunctival epithelial cells were present in stratified layers on the surface of the scaffolds. The number of layers increased over time, ranging from 1–2 layers at day 3 to 3–6 layers at day 14. Epithelial cell morphology was similar to that of control cells grown on conventional plastic culture surfaces [21], with characteristic polygonal shape. In contrast, fibroblasts grown inside the matrices had the typical elongated shape of this cell type.

### Epithelial cells maintained phenotype when cultured on fibrin scaffolds

A problem that often occurs when culturing epithelial cells is epithelial-mesenchymal transition (EMT) [22]. To assure that the epithelial cells cultured in the matrices were not undergoing EMT, the conjunctival epithelial marker CK19 was analyzed on different days. Cultured epithelial cells on both plasma and cryoprecipitate matrices expressed CK19 at all times, from day 3 to day 14 (Fig 2b).

In addition, the presence of mucin-secretory cells was analyzed using the lectin HPA. HPA-stained cells were observed at days 3 and 7, but almost none at day 14 (Fig 2c). This finding indicates that goblet-like cells are present in the scaffolds for at least seven days.

### Cell proliferation in the fibrin scaffolds

**Fig 3:**
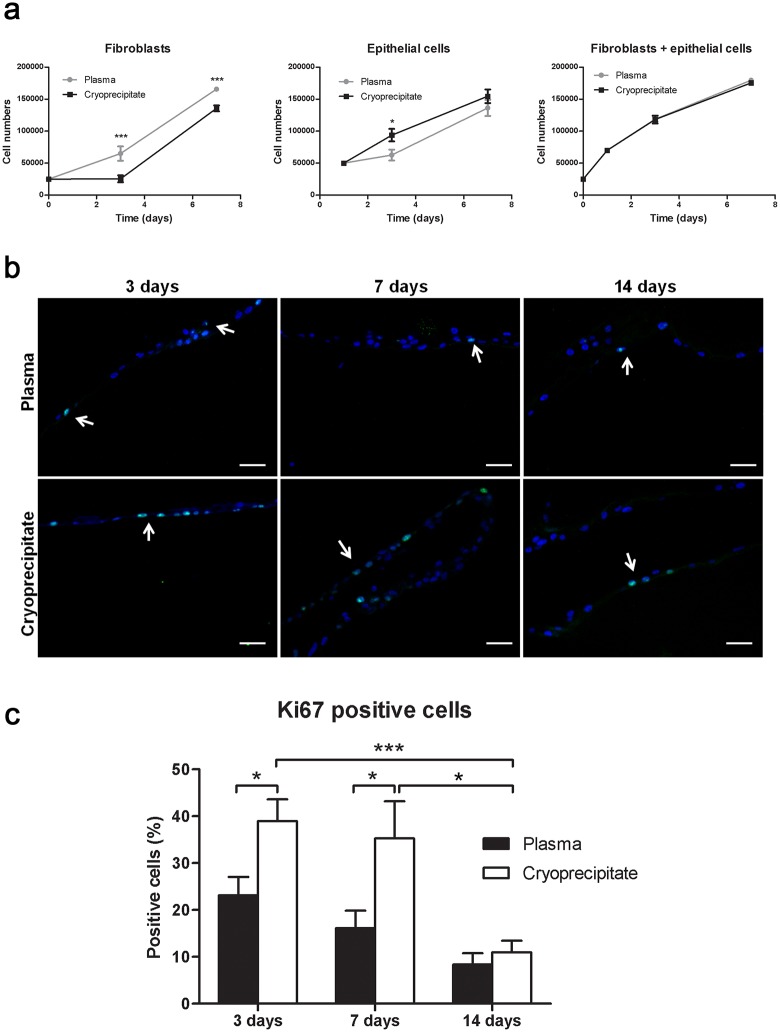
Conjunctival cells proliferated at different rates when seeded in plasma or cryoprecipitate scaffolds. Proliferation was measured by Ki67 staining (green). **(a)** Proliferation of fibroblasts alone (left), epithelial cells alone (middle), or both (right) was measured with alamarBlue^®^ assay in four independent experiments. *p ≤ 0.05; ***p ≤ 0.005. **(b)** Some Ki67-stained cells are indicated by arrows. Nuclei were stained with Hoechst dye (blue). Scale bar: 50 μm. **(c)** The percent of positive cells for the proliferation marker Ki67 was higher in cryoprecipitate scaffolds than in plasma ones at days 3 and 7. That percent was significantly decreased at 14 days. * p ≤ 0.05; *** p ≤ 0.005.

As determined by the alamarBlue^®^ assay, conjunctival cells retained proliferative capacity in the matrices. Fibroblasts proliferated better in plasma scaffolds, whereas epithelial cells had better growth rates in cryoprecipitate matrices (Fig 3a). When both cell types were analyzed together, there was no significant difference in the proliferation rates.

In addition, the expression of the proliferation marker Ki67 was analyzed in cell-seeded plasma and cryoprecipitate scaffolds to determine the growth capacity of epithelial cells at 3, 7, and 14 days. Several positively stained cells were present in both plasma- and cryoprecipitate-derived fibrin scaffolds at all studied times (Fig 3b). However, there were significant differences in the percentage of positive cells in each condition (Fig 3c). The number of positive cells was significantly higher in cryoprecipitate matrices compared to plasma matrices at days 3 (38.97 ± 4.63% *versus* 23.13 ± 3.94%, p = 0.012) and 7 (35.28 ± 7.92% *versus* 16.13 ± 3.72%, p = 0.023). In cryoprecipitate scaffolds, a significant decrease in the percentage of positive cells occurred over time, from 38.97 ± 4.63% at day 3 to 35.28 ± 7.92% at day 7, and 10.96 ± 2.48% at day 14 (day 14 *vs* day 3, p = 0.0004; day 14 *vs* day 7, p = 0.015).

### Epithelial cells and fibroblasts maintained cell morphology in the fibrin scaffolds

**Fig 4:**
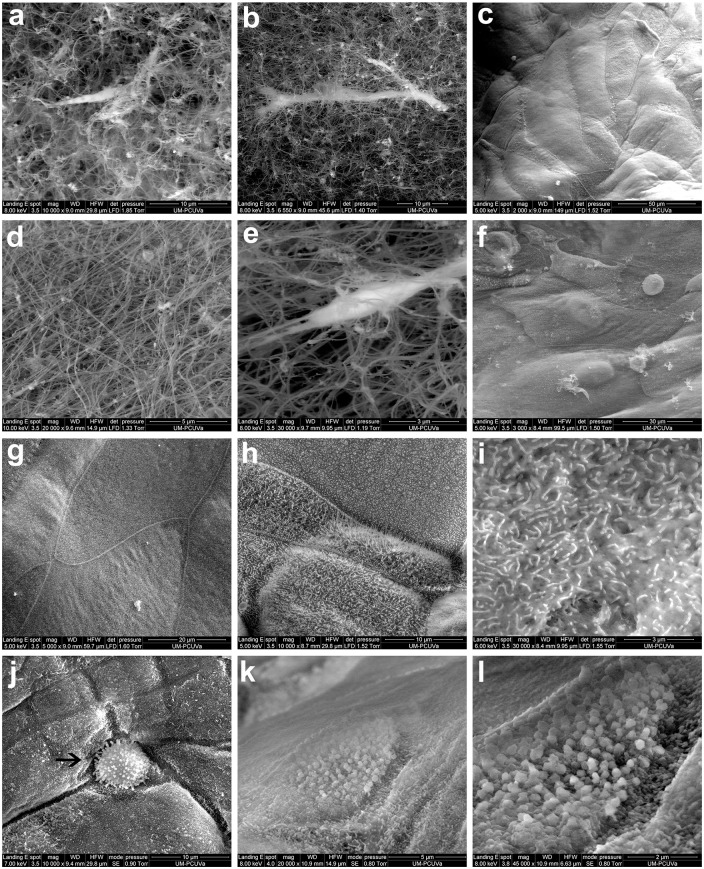
Scanning electron microscopy. Plasma **(a-c)** and cryoprecipitate **(d-f)** conjunctival constructs were observed by scanning electron microscopy. There was a higher density of fibrils in cryoprecipitate scaffolds **(d)**. Fibroblasts were integrated in the scaffolds **(b, e)** whereas epithelial cells covered the matrix surface **(c, f)**. Epithelial cells were tightly adhered to each other **(g)** and were covered by microvilli **(h)**, also shown in detail **(i)**. These features were present on the epithelial cells regardless of the whether the fibrin scaffold was produced from plasma or cryoprecipitate. Rounded cells, potentially goblet cells, were present **(j, arrow)**. Granules, potentially mucous granules, were also present **(k, l)**.

The scaffold structure and the morphology of fibroblasts and epithelial cells incorporated into both plasma (Fig 4a–4c) and cryoprecipitate matrices (Fig 4d–4f) were studied by SEM. The scaffold structure was similar in both cases, and fibrin fibrils were clearly observed in both cryoprecipitate and plasma matrices by SEM (Fig 4a and 4d).

Fibroblasts were evenly distributed inside the fibrin scaffolds and maintained elongated shape in both plasma (Fig 4b) and cryoprecipitate matrices (Fig 4e). Epithelial cells formed layers in which polygonal-shaped cells were identified in close contact with each other in both plasma (Fig 4c) and cryoprecipitate matrices (Fig 4f). The epithelial cells tightly adhered to each other, and cell borders were clearly distinguished from adjacent areas (Fig 4g). The entire surface of epithelial cells cultured on the surface of fibrin scaffolds was covered with microvilli (Fig 4h and 4i). Finally, goblet-like cells were present in the constructs (Fig 4j, arrow), and mucous secretion was observed (Fig 4k and 4l).

### Structural changes in response to experimental conditions

**Fig 5:**
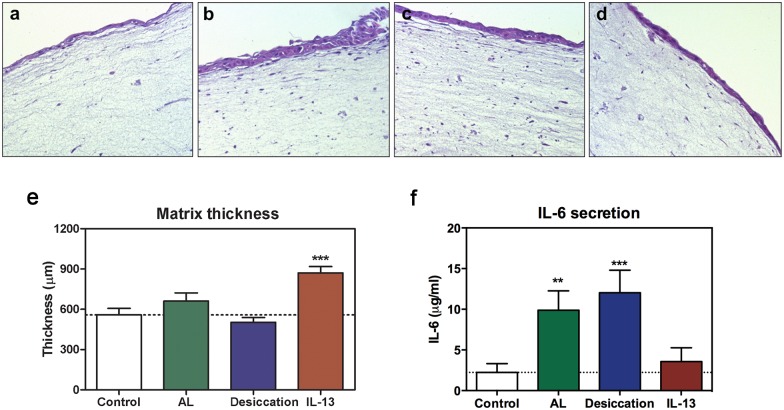
Constructs exposed to different experimental conditions showed different features. Hematoxylin/eosin sections of matrices in **(a)** control, **(b)** air-lifted, **(c)** partially desiccated, or **(d)** IL-13-treated conditions. Magnification: X20. **(e)** Matrix thickness was measured in the different experimental conditions. **(f)** The inflammatory cytokine IL-6 was secreted by conjunctival cells in the fibrin scaffold. In air-lifted (AL) and in partially desiccated cultures, IL-6 levels were significantly increased. Mean values from 6 independent experiments. **p ≤ 0.01; ***p ≤ 0.005.

Conjunctival constructs were fixed at day 7, processed, and stained with H/E to analyze cell distribution and potential structural changes (Fig 5a–5d). When constructs were cultured in air-lifted conditions for 3 days, the epithelium displayed a greater level of stratification (3–5 epithelial layers) compared to control (2 layers) cultures (Fig 5a and 5b). However, when air-lifted cultures were additionally exposed to 2 h of desiccation, the number of epithelial layers was reduced to 2 layers (Fig 5a–5c). No change in epithelial cell layers (2–3) was observed after IL-13 treatment (Fig 5d).

Fibrin matrix thickness in the four experimental conditions was measured in six independent experiments, and mean values were compared (Fig 5e). The thickness of control constructs was 558.12 ± 49.42 μm, whereas constructs treated with IL-13 for 24 h were significantly thicker, 871.75 ± 46.37 μm (p = 0.0008). Constructs desiccated for 2 h were slightly thinner, 503.22 ± 35.05 μm, than control constructs, although the difference was not statistically significant.

### Inflammatory responses

The levels of secreted inflammatory cytokine IL-6 were measured by ELISA to determine if the different experimental conditions produced an inflammatory response in the cells contained in the three-dimensional model (Fig 5f). Cells in the control constructs under basal conditions released 2.23 ± 1.08 μg/ml IL-6, and that secretion was significantly increased to 9.88 ± 2.37 μg/ml (p = 0.0088) and to 12.02 ± 2.77 μg/ml (p = 0.0040) in air-lifted and partially desiccated cultures, respectively. Constructs treated with IL-13 secreted 3.55 ± 1.70 μg/ml, which was not significantly different from levels secreted by control cultures.

### Effect of experimental conditions on mucous secretion

**Fig 6:**
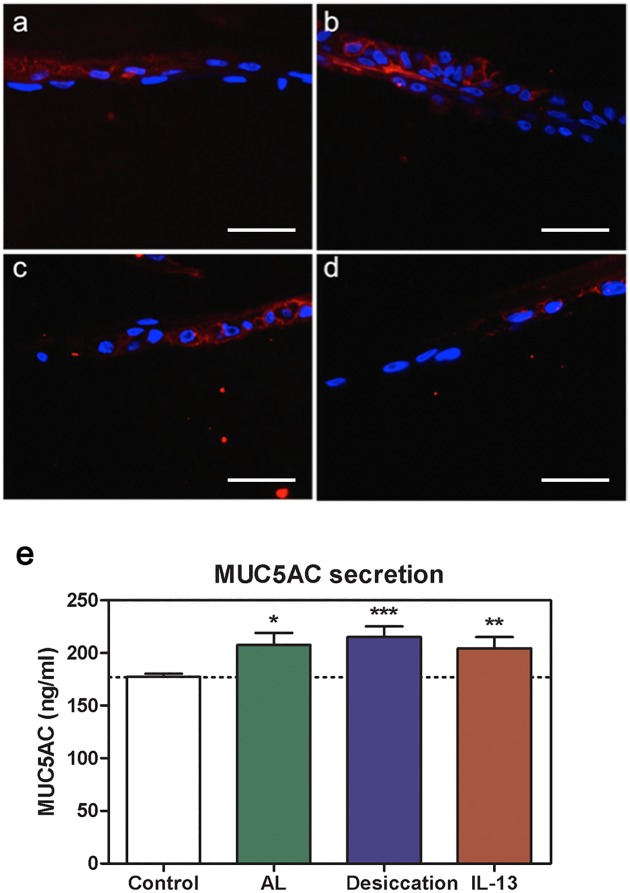
Mucin changes in the constructs. HPA staining (red) was detected in **(a)** control, **(b)** air-lifted, **(c)** partially desiccated, and **(d)** IL-13-treated constructs. Scale bar: 50 μm. **(e)** MUC5AC levels secreted by goblet cells present in the constructs were analyzed by ELISA. Each of the experimental treatments significantly increased secretion compared to control cultures. Mean values from 6 independent experiments. *p ≤ 0.05; **p ≤ 0.01; ***p ≤ 0.005.

The presence of mucus was evaluated in the constructs by two different techniques. First, constructs exposed to the four experimental conditions were stained with HPA (Fig 6a–6d). High molecular weight glycoconjugates identified by HPA were present in all of the experimental conditions. The number of stained cells increased in air-lifted cultures (Fig 6b). HPA staining was not altered by either partial desiccation for 2 h (Fig 6c) or IL-13 treatment for 24 h (Fig 6d).

In addition, mucin secretion in the constructs was evaluated by MUC5AC ELISA (Fig 6e). Secreted levels of MUC5AC increased from 177.37 ± 3.03 ng/ml in the control matrices to 207.54 ± 11.52 ng/ml in air-lifted cultures (p = 0.020). Under the partially desiccating conditions, secretion was 215.18 ± 10.23 ng/ml (p = 0.0026), and under IL-13 treatment, it was 204.44 ± 10.71 ng/ml (p = 0.0074). All of these findings suggest a modulation of mucin secretion by the *in vitro* goblet-like cells exposed to inflammatory conditions.

## Discussion

While there is a large amount of ongoing conjunctival research, there is a need to develop new methodologies that can reduce the reliance on animal use and accurately simulate the *in vivo* physiology of this tissue. In this study we engineered biocompatible scaffolds that contained primary cultures of fibroblasts and epithelial cells derived from human conjunctivas. These constructs maintained the conjunctival phenotype and retained biological function. In addition, we presented a proof of concept for its use to study ocular surface inflammation. We exposed the engineered conjunctiva to different conditions that mimic dry eye disease and allergic conjunctivitis, developing in this way a new three-dimensional *in vitro* model that could be used to test novel drugs against those prevalent diseases.

Different biomaterials are used in tissue engineering. Amniotic membranes have been widely used in the reconstruction of the ocular surface [23–25]. More recently, other biomaterials, such as collagen [26–28], gelatin [29], and fibrin [30,31] have also been tested for use in regenerative medicine. Fibrin is a natural biopolymer that is derived from the plasma protein fibrinogen. It is biocompatible, degradable, and promotes cell adhesion, which makes it an excellent material for tissue engineering [32]. It has been used as a scaffold to engineer dermis [33], cornea [34,35], and even rabbit conjunctiva [18]. Based on the success of these studies, we have used fibrin to develop the new three-dimensional model described here.

Although fibrin has been widely used as a scaffold in tissue engineering [30,31,36], to the best of our knowledge this is the first time that two types of fibrin-based matrices, derived from human plasma or cryoprecipitate, have been compared. Our results revealed differences in the interactions of conjunctival stromal and epithelial cells with the two types of matrices. While epithelial cells grew better in cryoprecipitate matrices, fibroblasts did it better in the ones derived from plasma. The fibroblasts were incorporated inside the fibrin scaffolds where the spacing in the plasma-derived matrices may be more supportive of proliferation for this type of cell. In contrast, epithelial cells showed the best results on cryoprecipitate matrices. Cryoprecipitates are obtained by the centrifugation of fresh frozen plasma. The product is more concentrated, especially with respect to the protein content, than plasma [37]. Furthermore, epithelial cells are more demanding in terms of nutrients and are more difficult to grow *in vitro* than fibroblasts [38]. Thus, the greater availability of plasma components in cryoprecipitates could be the reason why epithelial cells exhibited higher proliferation rates on this type of matrix.

Conjunctival epithelial cells developed into stratified layers on the surface of fibroblast-seeded fibrin-based scaffolds. This polarization is essential to mimic a normal conjunctival epithelium. Other authors have also described conjunctival primary cell stratification *in vitro*, but their cultures were limited to the epithelium [20,39–41].

The epithelial cells in the scaffold constructs maintained CK19 staining up to 14 days, showing that the cells grown on the surface of the matrices kept their epithelial phenotype without exhibiting EMT. Epithelial cells in culture often show EMT, characterized by loss of epithelial cell markers, such as cytokeratins and E-cadherin, upregulation of mesenchymal markers such as vimentin or FSP-1, and acquisition of fibroblast-like cell shape [22]. When this happens, cells are no longer actual epithelial cells and transform to a fibroblastic phenotype [42]. Therefore, results obtained from cultures showing EMT are not completely reliable.

An important function of the conjunctiva is to secrete mucins that lubricate and protect the ocular surface. Conjunctival goblet cells in the ocular surface are specialized for secretion of MUC5AC. These cells can be identified with HPA that stains high molecular weight glycoconjugates contained in the goblet cell secretory products. In our constructs, we found some cells that stained with HPA at days 3 and 7, in both plasma and cryoprecipitate matrices. However, there was no HPA staining at 14 days. There could be two explanations to this finding. Goblet cells may have secreted all of their content by 14 days. Then, even if goblet cells were present in the culture, they could not be identified with HPA staining. The other possibility is that the goblet cells that were present at 7 days died before 14 days. Goblet cells are difficult to culture, and for this reason it is plausible that they were not able to grow or be maintained in the three-dimensional culture for a long time. In fact, the large majority of stratified conjunctival cultures do not contain goblet cells [39,40]. Goblet cell loss is a hallmark of squamous metaplasia, a pathological transition to a non-secretory, keratinized epithelium. Tan *et al*. [43] maintained conjunctival explants *in vitro* for several days and studied the appearance of squamous metaplasia. They observed a reduced number of goblet cells at eight days, and a complete loss after 12 days in culture. Those results are in agreement with the findings observed in our construct. Thus, our three-dimensional model used at longer periods of time could be a useful tool to study squamous metaplasia *in vitro*. Further research using different culture media and supplements and analyzing the fate of goblet cells between seven and 14 days would help to elucidate the observed reduction in goblet cell numbers.

The morphological study performed by SEM revealed that fibroblasts maintained an elongated shape and interacted with the fibrin, while epithelial cells completely covered the matrix surface. Well-formed microvilli were clearly identified on the epithelial cells, which means the cells were polarized similar to the native tissue. Cell polarity is essential for the function of epithelial tissues, including the conjunctiva [44]. Alteration in conjunctival epithelia microvilli occurs in several diseases, such as Sjögren’s syndrome, graft versus host disease, and other pathologies [45,46]. In addition, changes in the number of microvilli detected by SEM have been proposed as a method to identify alterations in conjunctival epithelium [47]. Finally, we observed cells having a morphology compatible with that of goblet cells. In addition, we found mucus on the surface of those cells. These SEM results corroborate the presence of goblet-like cells found with HPA staining.

Epithelial cells growing on the surface of the fibrin scaffold showed higher stratification when cultured in the air-lifted condition. The ability of epithelial cells to stratify when cultured in those conditions is in agreement with previous publications. Chung *et al*. [20] reported human conjunctival epithelial cell stratification and de Borja Callejas *et al*. [48] reported similar results in other airway epithelia. Thus, these results corroborated that epithelial cells in our three-dimensional model are able to respond as they were expected to do.

Conjunctival mucin secretion becomes altered in ocular surface inflammatory diseases, and we found similar changes in our experimental conditions. Goblet cells were present in all of the conditions, and the number increased in the air-lifted cultures. Again, this response is in agreement with previous publications by several authors in other mucosal epithelium models [49,50]. In addition, we measured MUC5AC secretion by cells in the constructs in all conditions. In the *in situ* conjunctiva, MUC5AC is secreted exclusively by goblet cells. Therefore, our results confirm the presence of goblet cells in the three-dimensional model. Moreover, there was an increase in MUC5AC secretion in air-lifted, partially desiccated, and IL-13-treated conditions compared to control constructs. Similar results were observed when the air-lifted culture was additionally exposed to IL-13. Our results are consistent with those of De Paiva *et al*. [51] and Contreras-Ruiz *et al*. [52] who reported elevated MUC5AC secretion in murine goblet cells exposed to IL-13.

In dry eye disease many molecules are overexpressed. One of those is IL-6, a cytokine that has been used in several studies as a marker of inflammation. Overexpression of IL-6 in dry eye patients correlates with the symptomatic severity of disease [53]. In our research, we measured secreted levels of IL-6 and found that in the air-lifted and in the partially desiccating conditions IL-6 levels were significantly increased, which is in accordance with Zhang *et al*. [53]. Overexpression of IL-6 is indicative of an inflammatory status of the cells in the constructs, and it supports the idea of using this model to study ocular surface inflammatory disease. Specifically, IL-6 is found elevated in conjunctival cytology samples from patients with dry eye disease [53], and the highest levels of IL-6 in our model were found in the partially desiccating condition, which mimicked dry eye inflammation.

In summary, we have developed a structurally and physiologically relevant conjunctival model to study inflammatory diseases. We have shown that by exposing the constructs to partial desiccation for just two hours it is possible to reproduce some of the features found in dry eye disease. Furthermore, treating the conjunctival constructs with IL-13 mimics some of the responses found in allergic diseases. Bearing all this in mind, we conclude that this three-dimensional model is functional, responds to several stimuli, and therefore it can be used to study ocular inflammatory diseases.
